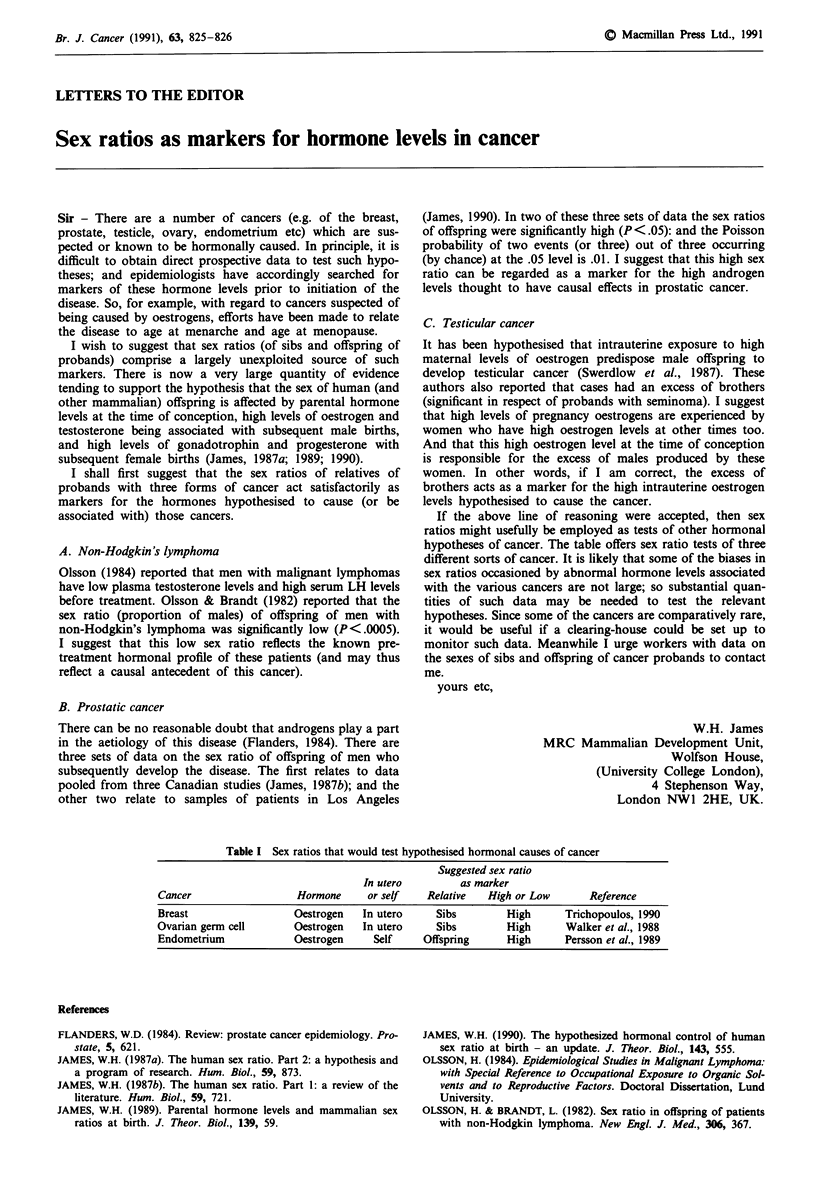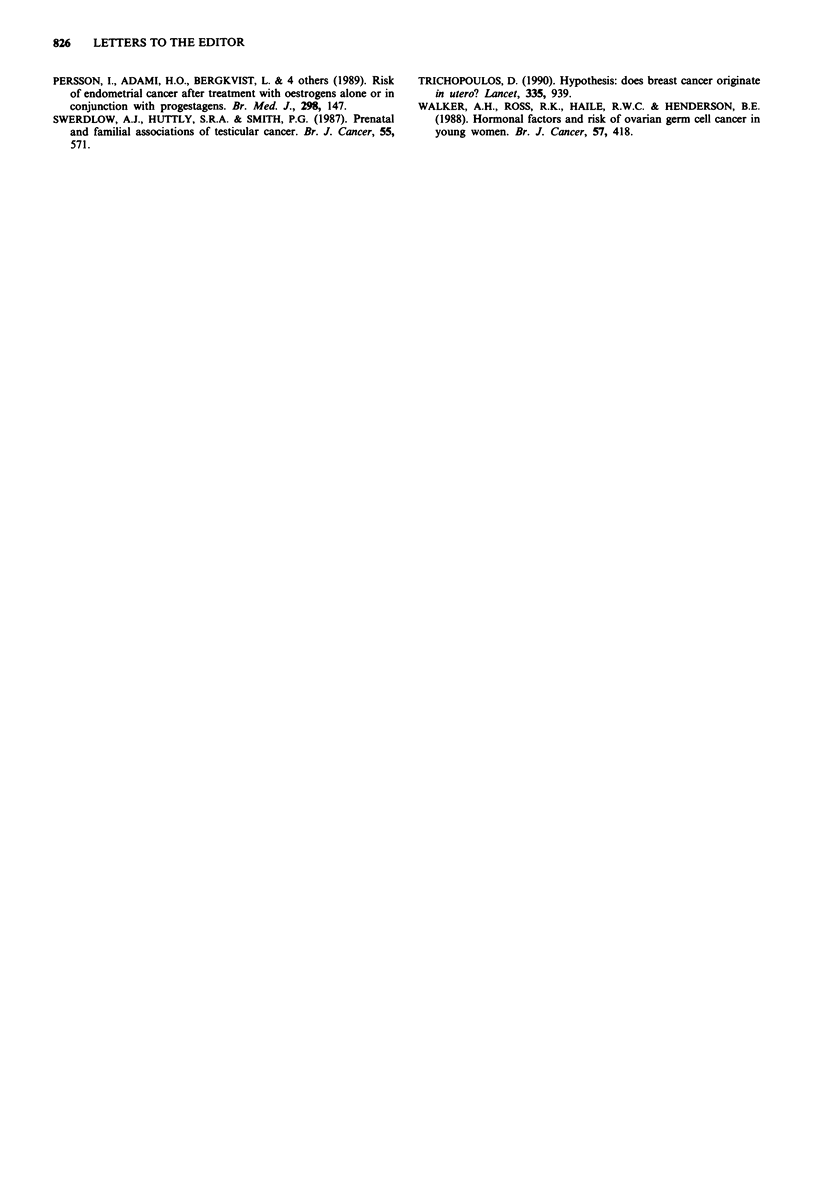# Sex ratios as markers for hormone levels in cancer.

**DOI:** 10.1038/bjc.1991.183

**Published:** 1991-05

**Authors:** W. H. James


Br. J. Cancer (1991), 63, 825-826 © Macmillan Press Ltd., 1991

### LETTERS TO THE EDITOR

### Sex ratios as markers for hormone levels in cancer

**Sir** – There are a number of cancers (e.g. of the breast, prostate, testicle, ovary, endometrium etc) which are suspected or known to be hormonally caused. In principle, it is difficult to obtain direct prospective data to test such hypotheses; and epidemiologists have accordingly searched for markers of these hormone levels prior to initiation of the disease. So, for example, with regard to cancers suspected of being caused by oestrogens, efforts have been made to relate the disease to age at menarche and age at menopause.

I wish to suggest that sex ratios (of sibs and offspring of probands) comprise a largely unexploited source of such markers. There is now a very large quantity of evidence tending to support the hypothesis that the sex of human (and other mammalian) offspring is affected by parental hormone levels at the time of conception, high levels of oestrogen and testosterone being associated with subsequent male births, and high levels of gonadotrophin and progesterone with subsequent female births (James, 1987*a*; 1989; 1990).

I shall first suggest that the sex ratios of relatives of probands with three forms of cancer act satisfactorily as markers for the hormones hypothesised to cause (or be associated with) those cancers.

*A. Non-Hodgkin's lymphoma*

Olsson (1984) reported that men with malignant lymphomas have low plasma testosterone levels and high serum LH levels before treatment. Olsson & Brandt (1982) reported that the sex ratio (proportion of males) of offspring of men with non-Hodgkin's lymphoma was significantly low ($P < .0005$). I suggest that this low sex ratio reflects the known pre-treatment hormonal profile of these patients (and may thus reflect a causal antecedent of this cancer).

*B. Prostatic cancer*

There can be no reasonable doubt that androgens play a part in the aetiology of this disease (Flanders, 1984). There are three sets of data on the sex ratio of offspring of men who subsequently develop the disease. The first relates to data pooled from three Canadian studies (James, 1987*b*); and the other two relate to samples of patients in Los Angeles (James, 1990). In two of these three sets of data the sex ratios of offspring were significantly high ($P < .05$): and the Poisson probability of two events (or three) out of three occurring (by chance) at the .05 level is .01. I suggest that this high sex ratio can be regarded as a marker for the high androgen levels thought to have causal effects in prostatic cancer.

*C. Testicular cancer*

It has been hypothesised that intrauterine exposure to high maternal levels of oestrogen predispose male offspring to develop testicular cancer (Swerdlow *et al.*, 1987). These authors also reported that cases had an excess of brothers (significant in respect of probands with seminoma). I suggest that high levels of pregnancy oestrogens are experienced by women who have high oestrogen levels at other times too. And that this high oestrogen level at the time of conception is responsible for the excess of males produced by these women. In other words, if I am correct, the excess of brothers acts as a marker for the high intrauterine oestrogen levels hypothesised to cause the cancer.

If the above line of reasoning were accepted, then sex ratios might usefully be employed as tests of other hormonal hypotheses of cancer. The table offers sex ratio tests of three different sorts of cancer. It is likely that some of the biases in sex ratios occasioned by abnormal hormone levels associated with the various cancers are not large; so substantial quantities of such data may be needed to test the relevant hypotheses. Since some of the cancers are comparatively rare, it would be useful if a clearing-house could be set up to monitor such data. Meanwhile I urge workers with data on the sexes of sibs and offspring of cancer probands to contact me.

yours etc,

W.H. James
MRC Mammalian Development Unit,
Wolfson House,
(University College London),
4 Stephenson Way,
London NW1 2HE, UK.

**Table I** Sex ratios that would test hypothesised hormonal causes of cancer

| Cancer | Hormone | In utero or self | Suggested sex ratio as marker: Relative | Suggested sex ratio as marker: High or Low | Reference |
|---|---|---|---|---|---|
| Breast | Oestrogen | In utero | Sibs | High | Trichopoulos, 1990 |
| Ovarian germ cell | Oestrogen | In utero | Sibs | High | Walker *et al.*, 1988 |
| Endometrium | Oestrogen | Self | Offspring | High | Persson *et al.*, 1989 |

**References**

FLANDERS, W.D. (1984). Review: prostate cancer epidemiology. *Prostate*, **5**, 621.

JAMES, W.H. (1987*a*). The human sex ratio. Part 2: a hypothesis and a program of research. *Hum. Biol.*, **59**, 873.

JAMES, W.H. (1987*b*). The human sex ratio. Part 1: a review of the literature. *Hum. Biol.*, **59**, 721.

JAMES, W.H. (1989). Parental hormone levels and mammalian sex ratios at birth. *J. Theor. Biol.*, **139**, 59.

JAMES, W.H. (1990). The hypothesized hormonal control of human sex ratio at birth – an update. *J. Theor. Biol.*, **143**, 555.

OLSSON, H. (1984). *Epidemiological Studies in Malignant Lymphoma: with Special Reference to Occupational Exposure to Organic Solvents and to Reproductive Factors*. Doctoral Dissertation, Lund University.

OLSSON, H. & BRANDT, L. (1982). Sex ratio in offspring of patients with non-Hodgkin lymphoma. *New Engl. J. Med.*, **306**, 367.

PERSSON, I., ADAMI, H.O., BERGKVIST, L. & 4 others (1989). Risk of endometrial cancer after treatment with oestrogens alone or in conjunction with progestagens. *Br. Med. J.*, **298**, 147.

SWERDLOW, A.J., HUTTLY, S.R.A. & SMITH, P.G. (1987). Prenatal and familial associations of testicular cancer. *Br. J. Cancer*, **55**, 571.

TRICHOPOULOS, D. (1990). Hypothesis: does breast cancer originate *in utero*? *Lancet*, **335**, 939.

WALKER, A.H., ROSS, R.K., HAILE, R.W.C. & HENDERSON, B.E. (1988). Hormonal factors and risk of ovarian germ cell cancer in young women. *Br. J. Cancer*, **57**, 418.